# The gut mycobiome: a novel player in chronic liver diseases

**DOI:** 10.1007/s00535-020-01740-5

**Published:** 2020-11-05

**Authors:** Lu Jiang, Peter Stärkel, Jian-Gao Fan, Derrick Eugene Fouts, Petra Bacher, Bernd Schnabl

**Affiliations:** 1 Department of Medicine, University of California San Diego, MC0063, 9500 Gilman Drive, La Jolla, CA 92093 USA; 2 Department of Medicine, VA San Diego Healthcare System, San Diego, CA USA; 3 Cliniques Universitaires Saint Luc, Université Catholique de Louvain, Brussels, Belgium; 4 Department of Gastroenterology, Xinhua Hospital, Shanghai Jiao Tong University School of Medicine, Shanghai, China; 5 Department of Genomic Medicine, J. Craig Venter Institute, Rockville, MD USA; 6 Institute of Immunology, Christian-Albrechts-University of Kiel and UKSH Schleswig-Holstein, Kiel, Germany; 7 Institute of Clinical Molecular Biology, Christian-Albrechts-University of Kiel, Kiel, Germany

**Keywords:** Fungi, Microbiome, Fungome, Alcohol-associated liver disease, Non-alcoholic fatty liver disease

## Abstract

The human gut microbiome (bacteria, fungi, viruses, and archaea) is a complex and diverse ecosystem. It plays an important role in human health, but is involved in several intestinal and extraintestinal diseases. Most research to date has focused on the role of bacteria, while studies focusing on fungi (also referred to as “mycobiome” or “fungome”) are still in its infancy. In this review, we focus on the existing literature available about the gut mycobiome with an emphasis on compositional mycobiome changes associated with liver diseases, the impact on pathogenesis of disease, and its potential use as therapeutic targets. We also provide insights into current methodologies of studying mycobiome, and we highlight the interkingdom interactions in the context of disease and how they affect health of the host. Herein, by focusing on the gut mycobiome, this review provides novel insights and directions for liver research.

## Introduction

The human gastrointestinal tract harbors trillions of microbes such as bacteria, fungi, viruses, and archaea [1, 2]. The gut microbiome may contribute to host metabolism, health, and disease [3]. Over the past decades, extensive studies elucidated the role of bacteria in health and disease due to the higher abundance and the existence of more established reference databases [4]. In contrast, the fungal constituents of the microbiome, termed the mycobiome, are receiving less attention yet highly important in maintaining intestinal homeostasis and immunity [5]. Although fungi comprise less than 0.1% of microbial genes [6], fungal cells are 100 times larger in volume than many bacteria, thus representing a much larger biomass [7], and they may play key roles in maintaining microbial community structure and function [8]. Fungi are an ancient community group, and the fossil evidence suggests that they may be 460 million years old with the earliest fungal from Ordovician [9]. As for now, at least 99,000 species of fungi have been defined, and approximately 1,200 new species are being described every year [10]. Most fungi grow as hyphae, which are tubule-like structures measuring 2–10 μm in diameter and several centimeters in length. Fungal nuclei are found small in diameter with 6–12 chromosomes encoding for 6,000–18,000 genes [11]. Compared with bacteria, the healthy human gut mycobiome is lower in diversity and density, and it is dominated by *Saccharomyces, Malassezia*, and *Candida* [12, 13]. Studies of the human mycobiome have been focused on several body sites including skin [14], gut [15], lung [16], and oral cavity [17]. Sequencing of internal transcribed spacer (ITS) 1 region from oral cavity of 20 healthy individuals identified 74 culturable and 11 non-culturable genera, with genera including *Candida*, *Cladosporium*, *Aureobasidium*, an unidentified Saccharomycetales, *Aspergillus*, *Fusarium*, and *Cryptococcus* being the most frequent ones [17]. A second study using ITS sequencing described oral fungal composition in healthy subjects and patients with periodontal disease. Overall, at least 5 phyla were detected (Ascomycota, Basidiomycota, Glomeromycota, Chytridiomycota, and unclassified), and most of sequences were assigned to the phylum Ascomycota. Similarly, *Candida* and *Aspergillus* were the most abundant genera (detected in 100% participants). At species level, three species were observed in all participants including uncultured *Dikarya*, *Candida * spp., and *Aspergillus niger* [18]. Human lungs continually exchange 5–8 L/min of air that contain 10^2^–10^4^ fungal spores per cubic meter [19]. In healthy subjects, there was low fungal amplification, and the few fungal reads present in healthy lung samples comprised largely environmental agents such as *Davidiellaceae, Cladosporium*, and low abundances of *Aspergillus* [20]. In contrast, the human gastrointestinal tract harbors a variety of fungal genera. Sequencing of ITS1 region from 96 human fecal samples detected 66 genera. The most common genera were *Saccharomyces*, *Candida*, and *Cladosporium* [21]. The skin mycobiome in adults is dominated by *Malassezia* [22], and fungi are more diverse including organisms such as *Aspergillus*, *Epicoccum*, and *Phoma* in addition to *Malassezia* [23].

Like bacteria, fungi can be beneficial to host immunity, but they can also have deleterious effects under disease conditions. Among all fungal infections, superficial infections of the skin and nails are the most common diseases with ~ 1.7 billion individuals being affected worldwide [24]. Invasive fungal infections are less frequent yet are more deadly, affecting ~ 1.5 million people every year. More than 90% of fungal-associated mortality is caused by species from one of these four genera: *Candida, Cryptococcus, Aspergillus*, and *Pneumocystis* [25]. The gut mycobiome has recently been recognized as a novel and important player in the pathophysiology of intestinal and extraintestinal diseases [2]. It is known to have a profound influence in modulating local as well as peripheral immune responses [26]. Recent data highlight that some commensal fungal species, especially *Candida albicans* (*C. albicans*) and *Malassezia* spp., are potent inducers of antigen-specific T-helper cell responses in humans that are altered in inflammatory diseases, such as inflammatory bowel diseases (IBD) [27] and skin disorders [28], which thus may contribute to disease pathogenesis. The gut and liver have a bidirectional communication via the biliary system and portal vein [29]. Therefore, the gut microbiota and microbial products play critical roles in modulating liver functions. In this review, we summarize the current evidence that supports the association between gut mycobiome and various liver diseases (Table 1), such as alcohol-associated liver disease (ALD) and non-alcoholic fatty liver disease (NAFLD). We discuss recent advances in gut mycobiome research, current methodologies and challenges to study mycobiome, and the bacteria–fungi interactions in the context of disease.

**Table 1:** Gut mycobiome or fungal-derived products in chronic liver diseases

Disease	Species	Mycobiome alterations in diseased individuals	References
Alcoholic hepatitis	Human	Decreased fungal diversity Increased *Candida* (genus) and *C. albicans* Decreased *Penicillium* (genus) Decreased *Saccharomyces* (genus) Increased serum ASCA Increased fecal ECE1 gene	[43, 44, 81]
Ethanol-induced liver disease	Mouse	Increased total number of fecal fungi Increased plasma 1,3-β-D-glucan Disease exacerbation by candidalysin	[44]
Obesity	Human	Decreased fungal diversity (family level) Decreased *Zygomycota* (phylum), Decreased *Agaricomycetes* (class) and *Mucor* (genus) Increased *Tremellomycetes* (class)	[45]
PSC	Human	Increased fungal diversity in PSC (compared with IBD) Decreased Saccharomycetales (order), Saccharomycetes (class), Saccharomycetaceae (family), and *S. cerevisiae* (species)	[92]
Cirrhosis	Human	Decreased fungal diversity in cirrhosis Increased *Candida* in inpatients Decreased Basidiomycota/Ascomycota in inpatients vs. outpatients and controls	[46]

*C. albicans, Candida albicans,* *ASCA,* anti-*Saccharomyces cerevisiae* antibodies, *ECE1,* extent of cell elongation 1, *PSC,* primary sclerosing cholangitis, *IBD,* inflammatory bowel disease, *S. cerevisiae,* *Saccharomyces cerevisiae*

### The role of fungi in host health and immunological responses

Our microbiome is a complex ecosystem, which influences diverse host functions such as metabolism, vitamin synthesis, gut permeability and function, and immunity [30]. However, understanding the dynamics and how it is controlled are challenging. Foster et al. argued that hosts are under selective pressure to control their microbiota towards the retention of beneficial species [31]. Therefore, it is possible that mycobiota is also under the selection by the host in favor of its health [4]. A number of studies revealed that the gut mycobiome plays an important role in maintaining intestinal homeostasis and systemic immunity [32], while fungal dysbiosis is associated with local and distal inflammatory diseases [26, 33]. Fungi interact with the immune system via pathogen-associated molecular pattern (PAMPs) that are recognized by innate immune receptors and are often shared between different fungal species. Recent studies show that certain fungal species can provoke cellular adaptive immune responses represented by CD4+ T lymphocytes [34]. The type of T-cell response required to control different fungal species differs remarkably [35], which becomes evident in patients with different primary immunodeficiencies [36]. Especially, Th17 cells, a subset of T-helper cells characterized by producing interleukin (IL)-17A, IL-17F, IL-21, and IL-22, have been described to be of particular importance for protecting against fungi infection. IL-17 functions through activating the IL-17 receptor, which further induces other inflammatory cytokines, chemokines, and antimicrobial peptides to exert anti-fungal activity [37]. Interestingly, patients with an impairment in Th17 immunity mainly suffer from mucocutaneous candidiasis [38], while broader fungal susceptibilities are described in patients with innate immune defects [39]. Consistently, germ-free mice colonized with *C. albicans* developed robust Th17 response [40], and *C. albicans* is a major target of human Th17 cells that can be identified in virtually all individuals [27, 41]. Induction of *C. albicans*-specific Th17 responses in healthy humans seems to occur during homeostatic interaction with the host in the absence of obvious inflammation. This is in line with intestinal commensal *C. albicans* colonization in mice, where Th17 cells are induced in the gut and mesenteric lymph nodes in the absence of any fungal infection or excessive inflammation [40, 42]. *C. albicans*-specific T-cell responses also broadly modulate human anti-fungal Th17 immunity via propagating Th17 cells cross-reactivity to other fungal species, such as *Aspergillus fumigatus* (*A. fumigatus*) [27], that rather generates a tolerogenic regulatory T-cell (Treg) response in healthy humans [35, 43]. These cross-reactive Th17 cells expand in patients with asthma, chronic obstructive pulmonary disease (COPD), or cystic fibrosis, contributing to fungus-induced pathological inflammation in the lung [27]. Whether *C. albicans*-driven cross-reactive Th17 cells also contribute to other gut-distal diseases merits further investigation. Besides *C. albicans*, human Th17 responses have also been described to target other fungi, especially *Malassezia* spp.[27, 28]. *Saccharomyces cerevisiae* (*S. cerevisiae*) spores have been described to favor a Th17 inducing environment under in vitro conditions [44]. However, overall, only few microbial species and in particular *C. albicans* have been identified as potent inducers of Th17 responses in humans [27], suggesting a specialized interaction pattern with the human host.

In contrast to the homeostatic interaction, *Candida* overgrowth is linked to several diseases, including IBD [45–47], alcohol-associated liver disease (ALD) [48, 49], obesity [50], and liver cirrhosis [51], and also exacerbates experimental colitis in mice [47, 52, 53]. *Malassezia restricta* has also been linked to IBD. In a mouse model of colitis, it exacerbates disease severity by stimulating inflammatory responses via CARD9, a signaling adaptor for anti-fungal defense as well as a genetic risk factor linked to Crohn’s disease and ulcerative colitis [47, 54]. Intestinal inflammation and epithelial damage in IBD may enhance interaction of fungi and immune cells, since *C. albicans*-specific Th17 responses are increased in the blood of Crohn’s disease patients [27]. Anti- *S. cerevisiae* antibodies (ASCA), that are cross-reactive to *C. albicans,* can be detected in a subset of Crohn´s patients [55] and are also elevated in patients with ALD and associated with increased mortality in patients with alcoholic hepatitis [48].

Whether fungus-specific T-cell responses are altered in ALD is currently unknown. However, increased levels of Th17 cells and their cytokines are found in blood and livers of patients with chronic liver diseases [56–58], and recombinant IL-17A administration induces non-alcoholic steatohepatitis (NASH) in mice [57]. In ALD, elevated serum Th17 cytokines correlated with the progression of liver damage [58], while Th17 inhibition ameliorated experimental ethanol-induced steatohepatitis [59]. Future studies are needed to investigate a potential direct interaction between *C. albicans* and Th17-mediated pathology in chronic liver diseases.

Similar to bacteria, fungi can also affect host metabolism and health. For example, *S. cerevisiae* exacerbate colitis in a mouse model by enhancing host purine metabolism and increasing the systemic level of uric acid [60]. Specific *Malassezia* spp. can produce metabolites acting as virulence factors that promote inflammation and further contribute to diseases, while other metabolic products may downregulate inflammatory mediator production [61]. *Malassezia furfur* converts tryptophan into several indole compounds, including malassezin, indirubin, and indolo [3,2-b] carbazole (ICZ), as potent ligands for the aryl hydrocarbon receptor (AhR). AhR is a nuclear receptor expressed in all skin cell types, activation of which has been linked to skin homeostasis and certain skin diseases [61]. In healthy skin, AhR signaling contributes to keratinocyte differentiation, skin pigmentation, and skin barrier function. Several studies have shown that blocking AhR signaling prevents or treats skin cancer, whereas activating AhR could be beneficial in inflammatory skin diseases [62]. Therefore, AhR signaling could be a promising target for skin diseases. Taken together, these studies show the importance of fungi and their metabolites for host health and disease. Understanding their interactions will facilitate a better understanding of disease pathogenesis and identification of targets for new therapy.

### Methodologies to study the mycobiome

Historically, scientists have relied on culture-based methods to study the mycobiome by plating onto selective media supplemented with antibiotics to prevent bacterial growth [63]. Sabouraud dextrose media (SABDEX) supplemented with antibiotics (e.g., chloramphenicol, tetracycline, or gentamicin) is a commonly used media for culturing yeast, molds, and aciduric bacteria [2, 64, 65]. Potato dextrose media are also widely used for culturing molds and yeast [64, 66]. For culturing fast-growing fungal species, such as *C. albicans*, Yeast Extract–Peptone–Dextrose (YPD) media supplemented with antibiotics has been used [67]. Chromogenic media, CHROMagar™ Candida, have been developed for visual differentiation of *C. albicans*, *C. tropicalis,* and *C. krusei* [68]. Leeming and Notman agar can be used to culture *Malassezia* spp., which can be observed after incubation for 4 days [69]. Similar to what has been encountered for culturing bacteria and archaea, challenges exist for most fungal microorganisms [38]. For example, culture-based methods have a number of issues and biases, such as time-consuming, and they are biased towards fast-growing and non-fastidious species [2]. Previous studies reported that culture-based methods identified less than 30% of the fungal species from human gut compared to culture-independent methods, such as ITS1 and ITS2 sequencing [70].

To circumvent these challenges, a culture-independent method to investigate fungal communities is performing polymerase chain reaction (PCR) on extracted DNA using different primers [71]. Different regions of the fungal ribosomal DNA operon including 18S, 5.8S, 26S, and the ITS are targeted by different primers [72]. 18S region is the most widely used to study mycobiome and other eukaryotes, while it provides lower taxonomic resolution than ITS regions [73], and it might amplify non-fungal species from the host [64]. As the rapid development of next-generation sequencing, ITS regions located between 18S, 5.8S, and 28S, ITS1 and ITS2, are widely used for taxonomic identification of fungal species [73]. Despite the advantages of NGS on ITS sequencing, studies indicate that there are biases toward fungal species in terms of amplifying ITS1 or ITS2 regions [12, 32, 73]. Regardless of the methods used, high-quality reference databases are required to accurately assign fungal taxonomy [74]. Many fungal sequences submitted to GenBank have been annotated with incorrect species names, and more than 10% ITS sequences are annotated incorrectly at the species level [75]. There are three commonly used public fungal databases: the UNITE database [76], Findley database [77], and the RTL database [78]. As the choice of database will clearly affect the outcome of the study, it should be chosen with caution and tailed to the study.

### Mycobiome and alcohol-associated liver disease

Alcohol-associated liver disease (ALD) represents a spectrum of liver disease ranging from simple steatosis to the more advanced alcoholic hepatitis and cirrhosis resulting from alcohol use [79]. In the United States, ALD associated mortality was estimated 5.5 per 100,000 in 2012, and the contribution to cirrhosis is trending to increase compared with hepatitis C virus [80]. Early discoveries in animal models and patients associated with ALD identified increased level of bacterial endotoxin, disrupted intestinal barrier, and increased intestinal permeability and bacterial translocation [81, 82], suggesting a link between gut microbiome and the pathogenesis of ALD. Recent studies showed that alcohol consumption is associated with decreased bacterial diversity and altered gut microbiome in animal models and human patients [83, 84]. Furthermore, intestinal virome characterization showed an increased viral diversity in fecal samples from patients with ALD compared with nonalcoholic controls, and altered viral taxa are associated with disease severity and mortality in alcoholic hepatitis patients [85]. Research in the field of mycobiome showed changes in the composition of fecal mycobiome in patients with ALD compared with non-alcoholic controls. Patients with ALD exhibited lower fungal diversity and richness and a significant overgrowth of *Candida* (genus) independent of stages of ALD (nonprogressive ALD, alcoholic hepatitis, or alcoholic cirrhosis). In addition, there was an increased level of serum anti-*Saccharomyces cerevisiae* IgG antibodies (ASCA) in alcohol use disorder (AUD) patients with simple steatosis or progressive ALD [86] as well as in patients with alcohol-related cirrhosis compared with controls, indicating an increased systemic exposure to intestinal fungi or their products in response to alcohol. More importantly, serum ASCA level is correlated with mortality in patients with alcohol-associated cirrhosis or alcoholic hepatitis [48, 49]. Increased fungal overgrowth and plasma 1,3-β-D-glucan (cell wall polysaccharides from most fungi) were observed in mice following 8 weeks of chronic ethanol administration, compared with control diet-fed mice. Treating mice with anti-fungal agent amphotericin B reduced fungal overgrowth and 1,3-β-D-glucan translocation, and attenuated ethanol-induced liver disease via reducing C-type lectin-like receptor CLEC7A dependent IL-1β production [49]. *Candida albicans* (*C. albicans*) is a commensal fungus in the human gut, our previous study suggested that its relative proportion was increased in patients with AUD and alcoholic hepatitis patients. The proportion of patients with alcoholic hepatitis positive for fecal candidalysin, an exotoxin produced by *C. albicans*, is higher as compared with non-alcoholic controls or patients with alcohol use disorder. Candidalysin exacerbates ethanol-induced liver disease in mice by causing direct hepatocyte damage. Alcoholic hepatitis patients that are positive for candidalysin coding gene *ECE1* (extent of cell elongation 1) exhibit more severe disease and higher mortality [67]. Notably, candidalysin does not increase intestinal permeability or cause intestinal epithelial cell damage in ethanol-fed mice. It is possible that candidalysin reaches the liver from intestinal lumen via increased intestinal permeability [67]. Candidalysin can directly damage hepatocytes in culture.

The genus *Penicillium* dominated the mycobiome of nonalcoholic controls, and the abundance was negatively correlated with inflammatory grade and Mallory–Denk bodies on liver biopsy in patients with alcoholic hepatitis [48]. Whether *Penicillium* plays a role in the pathogenesis of ALD remains to be studied in the future.

### Mycobiome in obesity and nonalcoholic fatty liver disease

Non-alcoholic fatty liver disease (NAFLD) is the most common cause of chronic liver disease worldwide with around 30% of the entire population being affected [87]. NAFLD is associated with obesity and often considered the liver manifestation of the metabolic syndrome, and many factors are involved in the development of this complex disease, such as over-nutrition, genetic, and environmental factors [88, 89]. The spectrum of NAFLD ranges from simple steatosis to nonalcoholic steatohepatitis (NASH), which can progress to cirrhosis and even hepatocellular carcinoma (HCC) [88]. Dysregulation of gut microbiome has been linked to the pathogenesis of NAFLD in animal models and human studies [29, 90, 91]. There are limited studies on the relationship between NAFLD and gut mycobiome. One study used ITS sequencing to evaluate fungal diversity and composition in fecal samples of obese and non-obese subjects. Obese patients exhibited lower fungal diversity at family level, while there was no difference at other taxonomic levels.

Importantly, obese patients have distinct fungal composition compared with non-obese subjects. For example, obese subjects have an increased presence of phylum Ascomycota, class Saccharomyces, and families Dipodascaceae and Saccharomycetaceae and, an increased relative abundance of class Tremellomycetes compared with non-obese subjects. In addition, fecal samples of obese subjects were most abundant in *Candida*, *Nakaseomyces*, and *Penicillium* genera, while *Mucor* was the most prevalent genus in non-obese subjects. Furthermore, the decreased relative abundance of *Mucor* genus in obese subjects was reversible after weight loss [50]. Whether changes in mycobiota increase the risk or even contribute to obesity development is not known. Another study characterized gut fungal changes using ITS2 sequencing in mice fed with high-fat diet. Mice fed with high-fat diet exhibited similar fungal diversity compared with standard chow diet. The abundance *of Alternaria, Saccharomyces, Septoriella*, and *Tilletiopsis* genera; *Saccharomyces cerevisiae*; and *Tilletiopsis washingtonensis* were higher in mice fed with standard chow diet [92]. Notably, the predominant colonizer and pathobiont *C. albicans* were detected in both groups with similar relative abundance. Strong diet-specific coabundance relationships were observed between bacteria and fungi, and the number of coabundance correlations was reduced in high-fat diet-fed mice.

The probiotic *Saccharomyces boulardii* (*S. boulardii*) has been widely used as probiotic in clinical practice. It functions by consuming oxygen, which produces an anaerobic environment and facilitates the growth of bifidobacterial and lactic acid bacteria while inhibiting pathogens [93, 94]. In mice with diet-induced diabetes and obesity, oral gavage of *S. boulardii* daily for 4 weeks reduced body weight, fat mass, hepatic steatosis, and inflammation. *S. boulardii* increased cecum weight and induced gut microbiota composition changes at the phylum, family, and genus levels [95]. Similarly, in a rat model of diet-induced steatohepatitis, oral gavage of *S. boulardii* (7.5X10^9^ CFU/kg/d) for 8 weeks reduced body weight, hepatic steatosis, endotoxemia, and inflammation. It also regulates the ratio of *Escherichia coli/Bacteroides* in the intestine of rats, indicating a potential interaction between bacteria and fungi [96]. It is currently unknown whether fungi contribute to NAFLD pathogenesis, which requires further research in the future.

### Mycobiome and primary sclerosing cholangitis

Primary sclerosing cholangitis (PSC) is a chronic cholestatic liver disease characterized by the development of biliary inflammation and fibro-obliterative lesions [97, 98]. Previous studies have implicated the role of gut microbiota and its metabolites in the pathogenesis of PSC and PSC-associated inflammatory bowel disease (IBD), which exists in the majority of PSC patients [99]. Although PSC is considered a rare disease in global population, it is associated with an increased risk of colorectal and hepatobiliary malignancy, even cholangiocarcinoma [100]. The gut microbiota and its metabolites have been implicated in the pathogenesis of PSC and PSC/IBD [101] as well as with disease severity and clinical outcome [102]. Studies on gut microbiome showed enriched level of *Veillonella, Streptococcus,* and *Enterococcus* in PSC patients [103]. Among these altered taxa, *Veillonella * spp. can produce amino oxidases such as vascular adhesion protein-1 (VAP-1) that facilitates adhesion of gut-trophic lymphocytes to liver endothelium in a substrate-dependent manner. Elevated level of circulating form of VAP-1 is increased in PSC patients, and it is associated with disease severity and clinical outcome [102]. As most studies focus on the gut bacteria in PSC pathogenesis, the role of fungi has not been extensively studied. A possible role of fungi is first implicated by a high prevalence of serum ASCA in PSC patients [104]. Furthermore, *Candida* spp. (*albicans*, *glabrata*, *tropicalis*, and undifferentiated) in bile identified by culture-based methods is associated with a poor prognosis in patients with PSC, and these patients need liver transplantation relatively soon [105]. Recently, a study investigated fungal changes in feces from patients with PSC changes by ITS2 sequencing. Interestingly, alpha diversity (Shannon and Chao1 indexes) showed no differences among healthy subjects, patients with IBD only, PSC and IBD, and PSC only. Beta diversity analysis revealed a clustering of samples according to the groups. Furthermore, patients with PSC had a higher ITS2/16S diversity ratio compared with IBD only patients, suggesting an increased fungi-to-bacteria diversity ratio. Overall, the fungal microbiota was dominated by two phyla: Ascomycota and Basidiomycota. PSC patients had increased proportions of *Exophiala* genus and Sordariomycetes class, while the proportion of *Saccharomycetaceae* family and *Saccharomyces cerevisiae* species were decreased [97]. In particular, *Saccharomyces cerevisiae* was also decreased in patients with active IBD, and it is shown to have anti-inflammatory effects by producing cytokine interleukin (IL) 10 [45]. *Exophiala* genus is involved in infections known as phaeohyphomycosis in immunocompromised hosts. One case report showed that *Exophiala dermatitidis* caused systemic infection mimicking PSC in a patient without immunodeficiency [106]. Another case report found that *Exophiala dermatitidis* infection leads to liver cirrhosis and intrahepatic bile duct dilation [107]. The above studies suggest an altered fungal composition with an altered fungi–bacteria correlation network in PSC [97]. Future studies are needed to elucidate the mechanistic role of fungi on PSC development, and in particular, whether fungi contribute to intestinal inflammation. A deeper understanding might pave the way for developing mycobiome-based biomarkers and therapeutic targets in patients with PSC.

### Mycobiome in cirrhosis and hepatocellular carcinoma

Cirrhosis is a leading cause of death worldwide resulting from chronic liver diseases such as ALD, viral hepatitis, and NAFLD [108]. One major reason for the mortality is related to gut originated infections [109]. Gut bacteria have been characterized in fecal samples from cirrhosis patients by 16S rDNA sequencing. In particular, *Bacteroides* genus was dominant in both cirrhosis and healthy groups, but the level was significantly decreased in cirrhosis group. *Veillonella, Streptococcus, Clostridium*, and *Prevotella* were enriched in cirrhosis group, while *Eubacterium* and *Alistipe*s were dominant in healthy groups [110, 111]. While cirrhosis is associated with infections, the role of fungi has been increasingly recognized. One study including 143 cirrhotics (outpatients and inpatients) and 26 controls investigated the bacteria and fungi signature changes and the fungal stability over time in response to antibiotic use. In this study, they found Bacteroidetes/Ascomycota ratio changes with cirrhosis severity. Those changes also can predict 90-day hospitalizations in patients with cirrhosis independent of disease severity, encephalopathy, and clinical biomarkers. Fungal diversity parallels bacterial diversity, and is decreased in cirrhosis compared with healthy controls and negatively correlated with model for end-stage liver disease (MELD) score. Antibiotic therapy reduced bacterial and fungal diversity in outpatients with cirrhosis, while proton pump inhibitor use did not affect fungal diversity [51]. Another study demonstrated that patients with cirrhosis secondary to chronic hepatitis B infection have increased fungal abundance and higher fungal diversity compared with patients with chronic hepatitis B, carriers, and healthy controls, suggesting a positive correlation between fungal diversity and disease severity [112].

Hepatocellular carcinoma (HCC) is the most common primary cancer of the liver and is the sixth most common cancer in western countries [113]. Previous studies showed that fecal bacterial diversity was increased from patients with cirrhosis to early HCC with cirrhosis, both of which were caused by chronic HBV infection [114]. By comparing NASH-induced cirrhosis with or without HCC, patients with HCC had increased level of *Bacteroides* and Ruminococcaceae, whereas *Akkermansia* and *Bifidobacterium* were decreased [115]. The role of gut mycobiome is being less recognized and mainly focused on the role of aflatoxin, a food contaminant and carcinogen produced by fungi (such as *Aspergillus flavus* and *Aspergillus parasiticus*), in the pathogenesis of HCC [116]. Aflatoxin B1 is the most abundant member of the family, and it has been shown to contribute to HCC development by being converted to the reactive intermediate AFB1-8, 9 epoxide (AFBO) through cytochrome–P450 enzymes, that binds to hepatocellular DNA [117, 118]. The resulting DNA adducts interact with guanine bases of DNA and cause mutation in the *p53* tumor suppressor gene that leads to HCC [118]. HBV and aflatoxin B1 are known to co-exist in countries with high incidence of HCC, suggesting a possible interaction between the two factors [119]. HBV infection may sensitize hepatocytes to the carcinogenic effects of Aflatoxin B1 directly or indirectly, but the exact mechanism is unknown [120].

### Fungi–bacteria interactions in human disease

Currently, fungi–bacteria interactions have gained attention due to their impact on human health. Most studies have been focusing on the interactions between *Candida* species and bacterial species, since they are the most common infectious species in humans, and *C. albicans* being the leading pathogenic species out of over 150 known species [121]. In the human gastrointestinal tract, the interaction between *C. albicans* and the Gram-positive bacterium *Enterococcus faecalis* (*E. faecalis*) promotes a synergistic and non-pathogenic association with the host [122]. Interestingly, previous studies found increased abundance of *C. albicans* and *E. faecalis* in patients with alcoholic hepatitis compared with alcohol use disorder and non-alcoholic controls [67, 83]. If interactions really drive the disease progression or are the result of chronic disease remains to be investigated. In addition, recent studies in murine model demonstrate the existence of interaction between *C. albicans* and *Clostridium difficile* (*C. difficile*) in the context of *C. difficile* infection. In particular, mice pre-colonized with *C. albicans* showed increased survival and expression of *Il17a* in colon in *C. difficile* infection. Although there was no difference in *C. difficile* spore and toxin production, the beneficial bacterial genera *Bifidobacterium* and *Akkermansia* were increased in *C. albicans* pre-colonized mice [123]. Therefore, the interactions between bacteria and fungi may serve as important targets or new therapeutic tools in the host health and disease.

## Conclusions and perspectives

As the impact of human microbiome receives increasing attention, the study of mycobiome in host health and disease is still at an early stage. Although a number of studies have emerged to characterize the fungal signature in animal models and human studies, it remains unclear whether changes in mycobiome are consequences of the disease process or play pathogenic roles in disease etiology. Especially the potential link between changes in the mycobiome and altered immune responses driving inflammatory liver diseases is currently largely unknown and requires further investigation. While we have discussed studies whereby fungi are involved in liver diseases, there is a clear gap in this field. To date, most studies in the field of liver disease research focus on characterizing fungal signatures in patients with ALD, PSC, and cirrhosis. Additional research is needed to understand the complex interactions between fungi and other groups of microorganisms (bacteria and viruses), and how the interactions affect the host. Targeting mycobiome by anti-fungals or by fecal microbiota transplantation would help us manipulate fungi and understand its functionality.

## Abbreviations

ALD: Alcohol-associated liver disease
AUD: Alcohol use disorder
NAFLD: Nonalcoholic fatty liver disease
NASH: Nonalcoholic steatohepatitis
PSC: Primary sclerosing cholangitis
HCC: Hepatocellular carcinoma
ITS: Internal transcribed spacer
PCR: Polymerase chain reaction
AhR: Aryl hydrocarbon receptor
CD: Crohn’s disease
PAMP: Pathogen-associated-molecular-pattern
ASCA: Anti-*Saccharomyces cerevisiae* antibodies
*S. cerevisiae*: *Saccharomyces cerevisiae*
CLEC7A: C-type lectin-like receptor 7a
*ECE1*: Extent of cell elongation 1
*S. boulardii*: *Saccharomyces boulardii*
VAP-1: Vascular adhesion protein-1
IL: Interleukin
IBD: Inflammatory bowel disease
MELD: Model for end-stage liver disease
HBV: Hepatitis B virus
*E. faecalis*: *Enterococcus faecalis*
C. *difficile*: *Clostridium difficile*
